# Addiction of tobacco chewing and smoking in the patients of head and neck squamous cell carcinoma: A descriptive epidemiological study in Pakistan

**DOI:** 10.12669/pjms.35.6.1309

**Published:** 2019

**Authors:** Madiha Kanwal, Ghulam Haider, Uzma Zareef, Saima Saleem

**Affiliations:** 1 Madiha Kanwal, PhD Student. The Karachi Institute of Biotechnology and Genetic Engineering (KIBGE), University of Karachi, Karachi, Pakistan; 2 Ghulam Haider, MBBS, FCPS (Medicine), FCPS (Oncology), Jinnah Postgraduate Medical Centre (JPMC), Karachi, Pakistan; 3 Uzma Zareef, BDS, MCPS, (Oral & Maxillo-Facial Surgery), Liaquat College of Medicine and Dentistry (LCMD), Karachi, Pakistan; 4 Saima Saleem, PhD. The Karachi Institute of Biotechnology and Genetic Engineering (KIBGE), University of Karachi, Karachi, Pakistan

**Keywords:** Epidemiology, Head and neck squamous cell carcinoma, Tobacco adducts

## Abstract

**Objective:**

The associated risk factors for the majority of patients with head and neck squamous cell carcinoma (HNSCC) are tobacco and betel nut abuse, while there also seems to be a rising proportion of patients who report no history of tobacco or betel nut usage. Therefore, objective of the study was to find out potential risk factors and demographics of HNSCC patients addicted to tobacco and/or betel nut, as well as non-addicted patients.

**Methods:**

This epidemiological study was conducted in Karachi Institute of Biotechnology and Genetic Engineering (KIBGE), University of Karachi, and Jinnah Postgraduate Medical Centre (JPMC) from January to December 2016. All subjects were participants in an epidemiological study of HNSCC. Demographics and clinical characteristics were obtained for 185 addicted and 26 non-addicted patients.

**Results:**

Non-addicted patients were more likely to be females (χ^2^=19.0, *p*<0.001) and were significantly younger than addicted patients (χ^2^=21.0, *p*<0.001). Addicted patients more likely belonged to a lower income group (χ^2^=14.4, *p*=0.006) and had a higher proportion of oral cancers (χ^2^=30.0, *p*<0.001). Almost all addicted females had oral cancers (97%), whereas more than half of the non-addicted females had oral cancers (53%).

**Conclusions:**

Addicted patients commonly have oral cavity cancers. This might be due to the habit of chewing tobacco and/or betel nut that addicted patients have. Non-addicted patients are commonly young females. It is likely that no single known factor is responsible for HNSCC in non-addicted patients, and several occupational exposure studies in future may be important to the etiology of non-addicted patients.

## INTRODUCTION

The annual incidence of head and neck cancers is more than 650,000 cases worldwide with approximately 330,000 deaths per year.^1^ Around 90% of all head and neck cancers are squamous cell carcinoma, known as head and neck squamous cell carcinoma (HNSCC).^2^ HNSCC is the sixth most common cancer worldwide^3^, but the second most common cancer in Pakistan.^4^ HNSCC is associated with various lifestyle and environmental risk factors. HNSCC has been strongly associated with tobacco use, the risk for HNSCC in habitual tobacco users is almost ten times higher than that in non-tobacco users. In fact, almost 70-80% of HNSCC diagnoses are associated with tobacco and alcohol use.^5^

Nearly 100 million people consume smokeless tobacco in Pakistan and India alone.^6^ Betel chewing, including betel quit and betel nut is common in South and Southeast Asia, and betel use also contributes to HNSCC cases.^7^ Habitual betel quit and betel nut use with or without added tobacco, has been classified as carcinogenic according to the International Agency for Research on Cancer (IARC).^8^ HNSCC in the developing countries vary from those in the Western world in terms of age at onset of the disease, site of disease, etiology, and genetic basis of the disease.

HNSCC cases without history of tobacco, alcohol and betel use were also observed during the sample collection of an ongoing molecular epidemiological study. This signifies the need to find out the possible risk factors for patients who never use tobacco, alcohol and betel in their lifetime, in order to prevent HNSCC.

## METHODS

This study was approved by the Institutional Review Board (IRB) at the Karachi Institute of Biotechnology and Genetic Engineering (KIBGE), University of Karachi (KIBGE/ICE/003/2015 Nov. 25, 2015), and Jinnah Postgraduate Medical Centre Karachi.

Patients included in this study were from the oncology department of JPMC from January to December 2016. To define the demographics and potential risk factors for HNSCC in tobacco and/or betel nut addicted and non-addicted patients, a total of 211 HNSCC diagnosed patients were included in this study.

A written informed consent was obtained from all the patients. Detailed demographic and socioeconomic information including age, gender, income, and education; information about betel nut and chewing tobacco consumption, cigarette smoking, alcohol consumption and other addictions were collected from the patients or their attendants. Clinical data containing HNSCC site, HNSCC subsite, TNM stage, and grade, were obtained by reviewing the patients’ medical record files in the hospital with the permission of the head of the department.

Patients were considered addicted if they had used betel nut or any tobacco products at least once a day for one year or more during their lifetime. Patients were considered non-addicted if they had never used betel nut, any of the tobacco products, and alcohol in their lifetime. Tobacco products included Paan, Gutka, Naswar, Manpuri, Cigarette, and Hookah.

Chi-square statistical tests were performed to compare addicted and non-addicted patient groups. Student’s t-test (independent samples t test) was used to compare age between the two groups and p ≤ 0.05 was considered as the level of statistical significance. All the statistical analyses were performed by using SPSS Statistics 17.0, Microsoft Excel and Tools for Science (online).

## RESULTS

In the current molecular epidemiological study database, 185 patients were identified as addicted while 26 were non-addicted. Ages of the patients were significantly different between the two groups. Non-addicted patients were significantly younger than addicted patients (Table-I). The non-addicted group had a higher proportion of patients being less than 40 years of age (42%), as compared to the addicted group (15%)

**Table I:** Demographic data for addicted and non-addicted patients with HNSCC.

Parameter	Addicted patient (n = 185)	Non-addicted patient (n = 26)		

	No. (%)	No. (%)	χ^2^ value	p value
***Age***			21.0	< 0.001^a^
< 40 y	28 (15.1)	11 (42.3)		
40-49 y	70 (37.8)	2 (7.7)		
50-59 y	38 (20.5)	9 (34.6)		
60-69 y	36 (19.5)	1 (3.8)		
> 69 y	13 (7.0)	3 (11.5)		
***Gender***			19.0	< 0.001^a^
Male	150 (81.1)	11 (42.3)		
Female	35 (18.9)	15 (57.7)		
***Income ^b^***			14.4	0.006^a^
< Rs.10,000	47 (31.5)	2 (18.2)		
Rs.10,000-Rs.19,000	87 (58.4)	7 (63.6)		
Rs.20,000-Rs.29,000	12 (8.1)	1 (9.1) 0 (0.0)		
Rs. 30,000-Rs.39,000	3 (2.0)			
>Rs. 40,000	0 (0.0)	1 (9.1)		
***Education***			9.55	0.049^a^
Uneducated	116 (62.7)	11 (42.3)		
8^th^ grade or less	39 (21.1)	8 (30.7)		
9^th^- 10^th^ grade	19 (10.3)	2 (7.7)		
11^th^- 12^th^ grade	7 (3.8)	2 (7.7)		
13^th^- 14^th^ grade or high	4 (2.1)	3 (11.5)		
***Mean age ± Std. Dev., y***	49.80 ± 12.04	44.08 ± 15.81		0.031^c^
***Median age, y***	48	47.50		
***Age range, y***	25-90	18-70		

*Abbreviations:* HNSCC: head and neck squamous cell carcinoma, Std. Dev.: standard deviation,

a Chi-square analysis,

b All female patients and 1 unemployed male patient were excluded,

c Student’s t test with Satterthwaite’s adjustment for unequal variances.

**Fig. 1:**
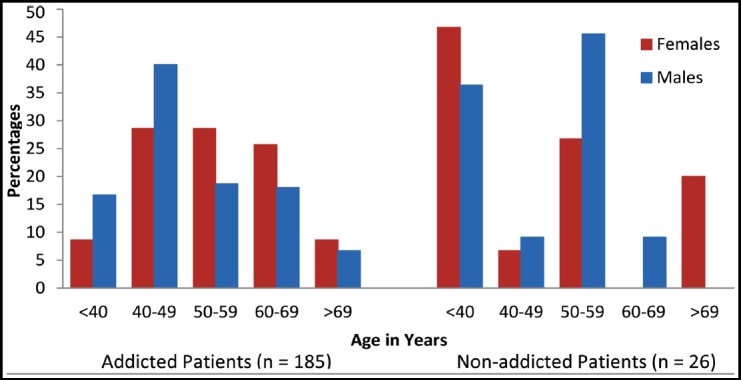
Age and gender distributions of addicted and non-addicted patients.

Females had a higher proportion in non-addicted patients group, while male patients were common in the addicted patients (Table-I and Fig. 1). A significant difference was observed in income between the groups. In addicted group, higher numbers of patients (90%) were found in Rs. 20,000/month income groups. A significant difference was also observed in the education; non-addicted patients were more educated than addicted patients. The HNSCC site distribution differed significantly among the two groups; addicted patients were more likely to have oral cancers and a higher proportion of laryngeal, and oropharyngeal cancers, while non-addicted patients had a higher proportion of hypopharyngeal, nasal cavity, eye, ear, and nasopharyngeal cancers. Subsite distributions of all sites of HNSCC were not significantly different between the two groups (Table-II).

**Table II:** HNSCC characteristics.

Parameter	Addicted patient (n = 185)	Non-addicted patient (n = 26)		

	No. (%)	No. (%)	χ^2^ value	p-value
***HNSCC site***			30.0	< 0.001
***Oral cavity***	157 (84.9)	15 (57.7)	12.5	0.132
Buccal mucosa	103 (65.6)	5 (33.3)		
Tongue	30 (19.1)	5 (33.3)		
Lip	7 (4.4)	0 (0.0)		
Gingival	5 (3.2)	2 (13.3)		
Palate	4 (2.5)	1 (6.7)		
Alveolar ridge	3 (1.9)	1 (6.7)		
Retromolar trigone	2 (1.3)	0 (0.0)		
Floor of mouth	1 (0.6)	0 (0.0)		
Buccal mucosa with any other subsite of oral cavity	2 (1.3)	1 (6.7)		
***Larynx***	14 (7.6)	1 (3.8)	1.22	0.747
Glottis	6 (42.9)	1 (100.0)		
Supraglottis	4 (28.6)	0 (0.0)		
Laryngeal wall	3 (21.4)	0 (0.0)		
Vocal cords	1 (7.1)	0 (0.0)		
***Hypopharynx***	6 (3.2)	3 (11.5)	6.00	0.112
Pyriform sinus	4 (66.7)	0 (0.0)		
Hypopharyngeal wall	1 (16.7)	2 (66.7)		
Cervical	1 (16.7)	0 (0.0)		
Post cricoid	0 (0.0)	1 (33.3)		
***Oropharynx***	2 (1.1)	0 (0.0)		
Tonsil	1 (50.0)	0 (0.0)		
Oropharyngeal wall	1 (50.0)	0 (0.0)		
***Nasal cavity***	1 (0.5)	1 (3.8)	2.00	0.157
Lateral wall	1 (100.0)	0 (0.0)		
Floor	0 (0.0)	1 (100.0)		
***Eye***	2 (1.1)	2 (7.7)	0.00	1.000
Orbit	1 (50.0)	1 (50.0)		
Eyelid	1 (50.0)	1 (50.0)		
***Ear***	2 (1.1)	1 (3.8)	2.00	0.157
Middle ear	2 (100.0)	0 (0.0)		
Auricle	0 (0.0)	1 (100.0)		
Nasopharynx	1 (0.5)	3 (11.5)		
***Differentiation^a^***			9.10	0.011
Well	52 (30.5)	1 (4.3)		
Moderate	111 (65.3)	19 (82.6)		
Poor	7 (4.1)	3 (13.0)		
***Stage^b^***			5.21	0.391
0	3 (3.1)	0 (0.0)		
I	1 (1.0)	0 (0.0)		
II	13 (13.3)	3 (30.0)		
III	36 (36.7)	5 (50.0)		
IV A	40 (40.8)	1 (10.0)		
IV B	5 (5.1)	1 (10.0)		

a Differentiation was not recorded for 15 addict and 3 nonaddict patients,

b Stage was not recorded for 87 addict and 16 non-addict patients.

**Fig. 2:**
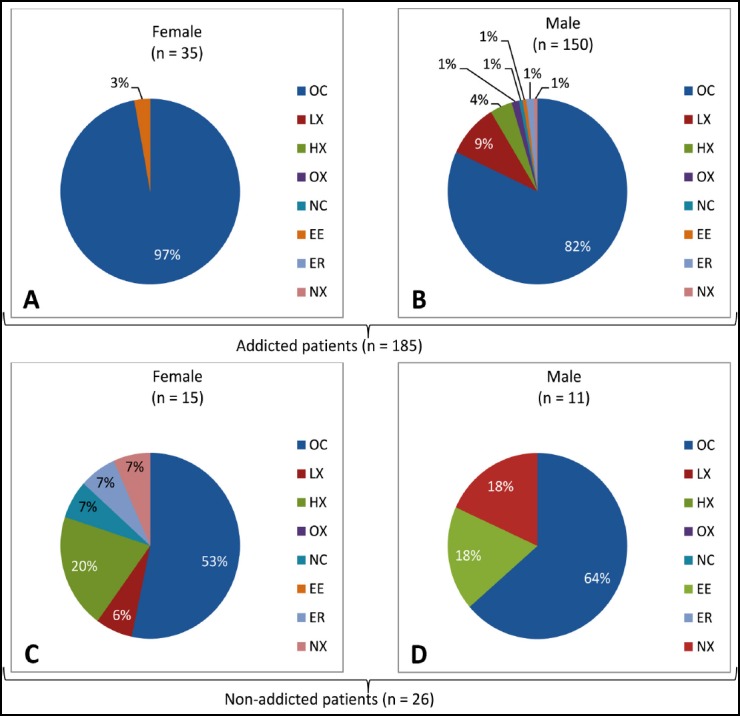
Cancer site distributions between male and female patients of addicted and non-addicted patient’s groups. OC, oral cavity; LX, larynx; HX, hypopharynx; OX, oropharynx; NC, nasal cavity; EE, eye; ER, ear; NX, nasopharynx.

However, differences were observed in HNSCC site distributions between male and female patients; 97% females and 82% males of addicted group had oral cancers (Figs. 2.A and 2.B), whereas in non-addicted patients, 53% of females and 64% of males had oral cancers (Figs. 2.C and 2.D).

The majority of addicted patients showed with either well-differentiated or moderately differentiated HNSCC, while most non-addicted patients showed with moderately differentiated HNSCC. Among addicted patients stage IV is commonly prevalent. Nevertheless, staging was not noted for a greater proportion of patients, so it is undecided whether staging was actually distributed differently between the groups.

## DISCUSSION

The findings revealed that HNSCC in addicted and non-addicted patients varies in many ways. In particular, patient age, gender, income, education, and HNSCC site of occurrence differ between the two groups of patients. These outcomes are important because clinicians need to know that HNSCC is not only restricted to betel nut and tobacco product users, but that people who have never used betel and tobacco products are also becoming an increasing proportion of HNSCC patients. In the long run, understanding these differences may be beneficial in the prevention and management of such cancers.

Other similar studies have also found that non-addicted HNSCC patients tend to be younger than addicted HNSCC patients.^9^ Similar to other studies, this study have also reported that non-addicted HNSCC patients are more likely to be females.^9^,^10^ An association of non-addicted patients with elderly females has been reported,^11^ but in this study non-addicted patients are being associated with young females.

Data used in this study is collected from a government hospital, where most of the patients are from rural areas. In rural households, the breadwinners are usually males, and females are housewives. Among non-addicted patients, more than half are females, who are housewives and do not earn money, so they cannot be included in the income categories. To eliminate this bias, females are excluded from the income groups. Higher numbers of addicted patients earn < Rs. 20,000/month. It has been reported that nicotine in tobacco reduces the appetite^12^, so people with low incomes might use tobacco products to suppress their appetites, ultimately leading to the development of HNSCC.

Similar to other studies,^4^,^13^-^16^ the oral cavity is the most common site of cancer development among all HNSCC patients, but some studies reported that larynx and hypopharynx,^17^ or oropharynx,^13^ are the most common sites of cancer. In addition, the oral cavity is also found to be the most common site of cancer occurrence in both addicted and non-addicted patient groups individually. It has also been found that HNSCC in either ever-smoker,^18^ or never-smoker patients are more likely to develop in oral cavity,^10^,^19^ but here addicted patients have greater proportion of oral cancers. In one study, the oropharynx is the most common site of HNSCC in never-smoker and never-drinker patients.^9^

The results also revealed a huge gender based difference, almost all addicted females (except one female) having oral cancers. Furthermore, almost half of the non-addicted females had oral cancers and half had other sites of cancers. Both genders have higher proportions of oral cancers in addicted patients as compared to non-addicted patients, which might be due the direct contact of betel nut and tobacco products to the oral cavity, which can enhance carcinogenic effects in oral cavity. The high proportion of oral cancers as compared to other sites in non-addicted patients might be due to familial history,^18^ HPV infections.^9^,^14^,^15^ or poor oral hygiene.^20^

Similar to other studies, most of the patients are presented with moderately differentiated cancers.^9^,^16^ Moreover, as 85% of addicted patients had oral cancers in this study, it is not surprising that when segregated by cancer site, in all three types of differentiation major proportions consist of oral cancers. Whereas in non-addicted patients, major proportion of only moderately differentiated cancers consist of oral cancers.

## CONCLUSIONS

This case series has documented the clinical characteristics and demographics of addicted and non-addicted HNSCC patients. Non-addicted patients with HNSCC are commonly young females. Addicted patients commonly have oral cavity cancers which might be due to the habit of chewing tobacco or betel nut in addicted patients. This study suggests that no single known factor is responsible for HNSCC in non-addicted patients; however, occupational exposure, environmental tobacco smoke, and family history of cancer may contribute to HNSCC development in non-addicted patients, which needs further investigation with large sample size.

### Authors’ Contribution:

**MK:** Concept and study design, analysis and interpretation of data, drafting the article, is responsible for integrity of research.

**GH& UZ:** Acquisition of data.

**SS:** Concept and study design, revising it critically for important intellectual content, final approval for publication.
